# Anxiety and depression in patients with single versus multiple keloids: A cross-sectional study

**DOI:** 10.1016/j.jdin.2026.05.011

**Published:** 2026-05-19

**Authors:** Lian Zhang, Yanyu Du, Dan Sun, PeiPei Huang, Renliang He, Bin Yang

**Affiliations:** Dermatology Hospital of Southern Medical University, Guangzhou, Guangdong, China

**Keywords:** anxiety, depression, keloids, psycho-dermatology

*To the Editor:* Keloids are disfiguring fibroproliferative disorders causing significant biopsychosocial burden.^1^ While the psychosocial impact is well-documented, the differential effect of lesion multiplicity, solitary versus multiple lesions, remains under-characterized. We evaluated whether keloid multiplicity independently predicts psychological morbidity.

In this cross-sectional study, 758 keloid patients (274 single, SK, versus 484 multiple, MK) were evaluated (Supplementary Fig 1, available via Mendeley at https://data.mendeley.com/datasets/bbwxbg8xxc/1). Psychological distress was measured using the Hospital Anxiety and Depression Scale (HADS), Self-Rating Anxiety Scale (SAS), and Self-Rating Depression Scale (SDS). Physical symptoms were assessed via the Vancouver Scar Scale (VSS; evaluating the most severe target lesion) and Visual Analog Scale (VAS) for global pain/pruritus.

The MK cohort exhibited significantly higher total HADS (10.55 ± 5.40 vs 9.38 ± 4.95, *P* = .015) and SDS scores (4.80 ± 3.01 vs 4.16 ± 2.89, *P* = .003) than the SK group (Table I, Supplementary Fig 2, available via Mendeley at https://data.mendeley.com/datasets/bbwxbg8xxc/1). To control for baseline demographic and clinical differences, multivariable linear regression was performed using SDS as the dependent variable. Adjusting for gender, age, disease duration, anatomical location, and prior treatments, disease multiplicity remained a statistically significant, independent predictor of higher depression scores (β = 0.547, *P* = .023; Fig 1).

**Table I:** Patient demographics and clinical characteristics

Characteristic	Single keloid (*n* = 274)	Multiple keloids (*n* = 484)
Age (y), mean ± SD	28.79 ± 8.68	27.40 ± 8.68
Gender (male/female)	104/170 (62.0% F)	311/173 (64.3% M)
Duration of Illness (y), mean ± SD	5.20 ± 4.83	5.91 ± 4.85
Trunk involvement, *n* (%)	196 (71.5%)	408 (84.3%)
Facial/neck involvement, *n* (%)	55 (20.1%)	100 (20.7%)
Previous treatment history, *n* (%)	274 (100.0%)	472 (97.5%)
VSS score, mean ± SD	10.78 ± 2.81	10.10 ± 3.14
VAS score, mean ± SD	4.73 ± 2.29	4.21 ± 2.08
HADS total, mean ± SD	9.38 ± 4.95	10.55 ± 5.40
SAS score, mean ± SD	5.23 ± 2.94	5.75 ± 3.17
SDS score, mean ± SD	4.16 ± 2.89	4.80 ± 3.01

**Fig 1:**
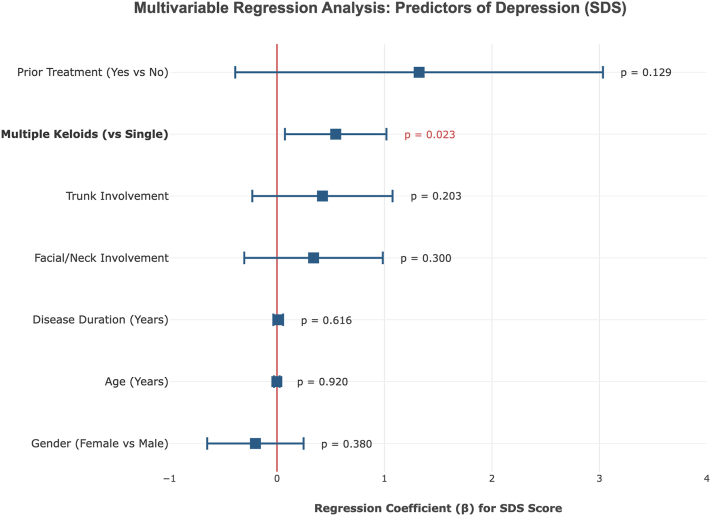
Multivariable regression analysis: Predictors of depression (SDS) in patients with keloid. A forest plot demonstrating the independent effects of demographic and clinical factors on Self-Rating Depression Scale (SDS) scores. Adjusted for gender, age, disease duration, anatomical location, prior treatment history, and disease multiplicity. Data are presented as regression coefficients (Beta) with 95% confidence intervals. The presence of multiple keloids is an independent predictor of higher depression scores (β = 0.547, *P* = .023).

Paradoxically, patients with SK reported significantly higher physical symptom severity (VSS: 10.78 ± 2.81 vs 10.10 ± 3.14; VAS: 4.73 ± 2.29 vs 4.21 ± 2.08; both *P* < .001) (Supplementary Fig 3, available via Mendeley at https://data.mendeley.com/datasets/bbwxbg8xxc/1). Correlation analysis revealed no association between physical symptoms (VSS/VAS) and psychological distress (HADS/SAS/SDS) in either group (r < 0.05) (Supplementary Fig 4, available via Mendeley at https://data.mendeley.com/datasets/bbwxbg8xxc/1).

These findings demonstrate that single and multiple keloids represent distinct clinical and psychological phenotypes.^2^ While existing literature frequently associates widespread or severe keloids with a higher prevalence of pruritus and pain, our cohort demonstrated a clinical paradox where SK patients reported higher localized physical symptom intensity despite a lower psychological burden. The exact mechanisms driving this dissociation remain unclear and warrant further keloid-specific investigation. Notably, anxiety was prevalent across both groups without an independent association with multiplicity (SAS *P* = .062), likely reflecting the universal burden of shared cosmetic concerns and social stigma.

Crucially, the uncoupling of physical severity from psychological distress challenges standard dermatological assessments that infer mental well-being from physical complaints. A patient with MK may report lower pain yet suffer from severe, silent depression. Therefore, we advocate for routine, independent psychiatric screening for patients with multiple keloids, recognizing lesion multiplicity as a critical determinant of depressive morbidity. Study limitations include the cross-sectional design and potential residual confounding from gender, as females generally report higher baseline body image and depression concerns in psychodermatology.^3^ Additionally, generic instruments like the HADS may not fully capture keloid-specific psychosocial issues (eg, stigma); future studies should employ targeted measures such as the SCAR-Q or the Dermatology Life Quality Index (DLQI).^4^

### Declaration of generative AI and AI-assisted technologies in the writing process

During the preparation of this work, the author(s) used DeepSeek to refine the English language and style. The author(s) reviewed and edited the output and take full responsibility for the content of the published article.

## Conflicts of interest

None disclosed.
